# Analyses of human cancer driver genes uncovers evolutionarily conserved RNA structural elements involved in posttranscriptional control

**DOI:** 10.1371/journal.pone.0264025

**Published:** 2022-02-25

**Authors:** Van S. Tompkins, Warren B. Rouse, Collin A. O’Leary, Ryan J. Andrews, Walter N. Moss

**Affiliations:** Roy J. Carver Department of Biophysics, Biochemistry and Molecular Biology, Iowa State University, Ames, IA, United States of America; Institute of Parasitology and Biomedicine, SPAIN

## Abstract

Experimental breakthroughs have provided unprecedented insights into the genes involved in cancer. The identification of such cancer driver genes is a major step in gaining a fuller understanding of oncogenesis and provides novel lists of potential therapeutic targets. A key area that requires additional study is the posttranscriptional control mechanisms at work in cancer driver genes. This is important not only for basic insights into the biology of cancer, but also to advance new therapeutic modalities that target RNA—an emerging field with great promise toward the treatment of various cancers. In the current study we performed an *in silico* analysis on the transcripts associated with 800 cancer driver genes (10,390 unique transcripts) that identified 179,190 secondary structural motifs with evidence of evolutionarily ordered structures with unusual thermodynamic stability. Narrowing to one transcript per gene, 35,426 predicted structures were subjected to phylogenetic comparisons of sequence and structural conservation. This identified 7,001 RNA secondary structures embedded in transcripts with evidence of covariation between paired sites, supporting structure models and suggesting functional significance. A select set of seven structures were tested *in vitro* for their ability to regulate gene expression; all were found to have significant effects. These results indicate potentially widespread roles for RNA structure in posttranscriptional control of human cancer driver genes.

## Introduction

Identification of cancer driver genes is an ongoing process [1, 2]. The ability to separate genes whose mutations are not directly responsible for the progress of neoplasticity (passengers) compared to genes whose mutations stimulate neoplasticity and malignancy (drivers) is important for future cancer therapeutic targeting. New computational methods are uncovering previously unappreciated oncogenes and tumor suppressors. One such recently developed method considered the nucleotide context in which mutational events occur to distinguish driver from passenger mutations. Dietlein et al. (2020) developed a method called Mutpanning [3], to identify driver genes based on a high number of mutational occurrences in unusual nucleotide contexts or outside mutational patterning normally observed for passenger mutations. The method does not require any prior knowledge of mutational functionality. Mutpanning alone identified 460 genes; some that were previously known and others that were previously unappreciated potential cancer driver genes. Another strategy is to combine several computational methods to identify both known and novel driver genes. Martinez-Jiménez et al. combined seven distinct methods, including Mutpanning, to identify 568 genes in their Integrative OncoGenomics compendium of drivers. About one-quarter of these had not been previously recognized in the Cancer Gene Census [4]. Combined, these methods point to 800 genes (228 in common, 232 unique to Mutpanning alone, 340 unique to the compendium) that have potential to drive cancer development and progression.

These driver gene identification methods focus on mutations in DNA coding regions that alter the protein output or quality–inducing a change in protein function in a context of time and space that enables acquisition of survival and proliferative advantages. Effects of non-synonymous mutations are relatively easy to understand because they directly affect the sequence of the protein. The role of synonymous and untranslated region (UTR) mutations in driver genes, however, are generally not well understood.

One step toward better understanding these driver genes is to enhance the knowledge of their RNA structure, which is known to play wide-ranging roles in posttranscriptional control mechanisms [5, 6]. We have developed a user-friendly computational tool to find structured regions in RNA that could potentially function in cellular homeostasis or in disease [7]. ScanFold makes use of a simple but powerful metric, a z-score that is rooted in thermodynamic stability of a given sequence of nucleotides. This z-score uses random sequence shuffling to relate the mean predicted minimum free energy (MFE) of random sequence folding to that of the native sequence, providing an estimated likelihood that the structure of the sequence with that native nucleotide *order* is more stable than by chance. That is to say, the z-score measures the unusual, ordered stability of RNA fragments, which can indicate that structure is an evolved property of that sequence. A further innovation of the ScanFold approach is that unique consensus model secondary structures are generated from recurring base pairs across low z-score regions, which tend to better reflect native folding [8]. We have shown that ScanFold is a reliable method for defining and modeling local RNA structural regions and have applied this approach to a variety of human genes including, most significantly to this current work, the *MYC* proto-oncogene, where an exceptionally stable motif was found that showed in vitro activity in regulating gene expression [9, 10].

While methods such as ScanFold can help to define regions of interest that may have evolved RNA structure/function, additional work is needed to validate those regions. One approach, which can also help to home-in on exceptionally interesting motifs, is analysis of covariation. Covariation model building [11–13] can be used to assess predicted structures against evolutionary mutations that preserve predicted base-pairing. Combined with statistical power analysis, the presence of covarying base pairs supports the presence of structured RNA [14] that has been selected over time, strongly suggesting a structure/function relationship. Together, thermodynamic predictions from sequence and historical evidence from covariation modeling boosts the likelihood of predicting functional RNA structures. Recently, we made use of these tools to identify structured regions of the SARS-CoV-2 virus [15], several of which have been explored as potential drug targets [16]—including one that was used to uncover a small-molecule inhibitor of viral gene expression [17].

Here, we apply our optimized RNA secondary structure discovery pipeline to transcripts of known and putative cancer driver genes. We describe a variety of extracted structural models with potential roles in posttranscriptional control and validate a small subset of select targets. The remainder provide a trove of potential targets for future studies (e.g. functional assays, drug targeting efforts); a resource we have organized and made publicly available.

## Materials and methods

### ScanFold

**Fig 1:**
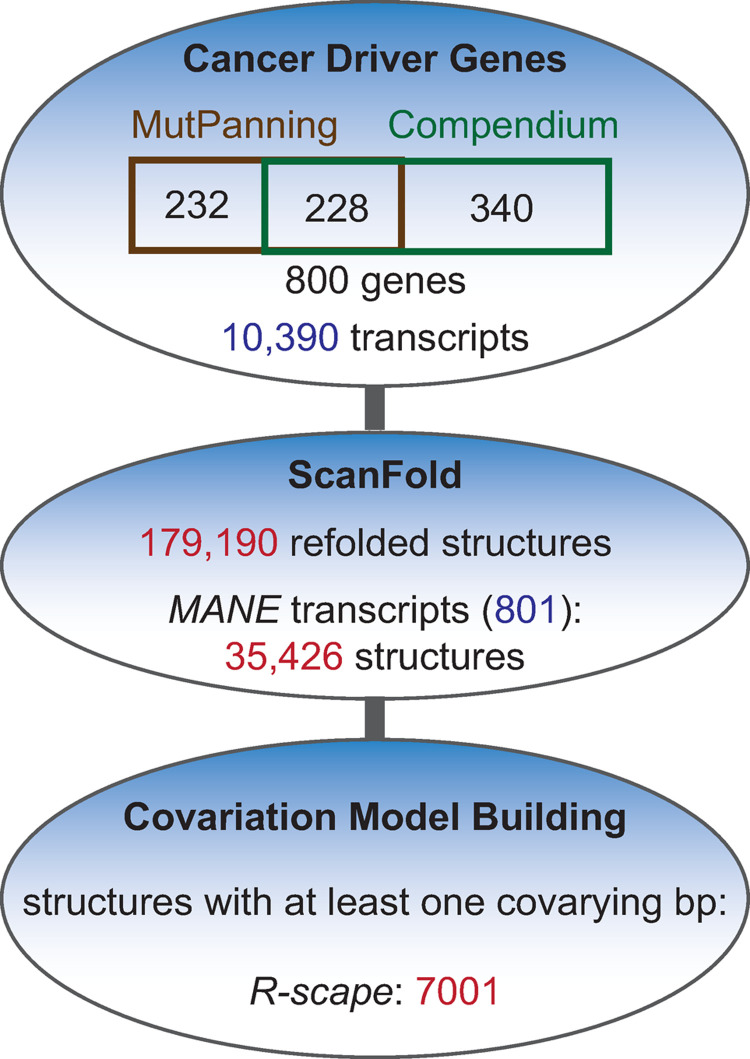
Flow diagram for study outline. Top oval shows the numbers of cancer driver genes and transcripts included in this study, as well as their sources–either from Mutpanning [3] or the Integrative OncoGenomics Compendium of drivers [4]. The gene symbols surrounding the flow diagram include all 800 genes in this study. The second oval shows the number of structures that were extracted from all the transcripts as well as from the matched annotation for NCBI and Ensembl (MANE) transcripts chosen for further analysis. The number of MANE structures exhibiting at least one covarying base pair (bp) after covariation analysis using structure-informed R-scape [12, 13] are shown in the bottom oval.

A list of 800 unique genes (Fig 1) was compiled using HGNC gene symbols from supplementary S3 Table of Dietlein et al. (2020) (460) and from the download section of the Integrative OncoGenomics website (https://www.intogen.org/) (568) [3, 4] (S1 Table). Ensembl BioMart (https://useast.ensembl.org/) was used to determine and download all Ensembl transcript identifiers (10,390 ENSTs) and sequences (Human GRCh38.p13) (S2 Table). All transcript sequences were analyzed using the ScanFold pipeline (ScanFold-Scan followed by ScanFold-Fold) with a single nucleotide step size, a 120-nucleotide window size, and 100 randomizations for z-score determination [7]. The z-score is the number of standard deviations from the difference in predicted minimum free energy (MFE) of base-pairing for a given sequence to the average MFE of 100 randomly arranged sequences of the same nucleotide composition [7]. All raw outputs (as described in [8]) are available at doi: 10.5281/zenodo.5747774. ScanFold determined all nucleotides with an average z-score of -1 or less from all windows containing that nucleotide. These were then constrained to base pair and the whole sequence was refolded using RNAfold [18]. A dot-bracket notation (dbn) file of this refolded (from Zavg_-1_pairs.dbn files) structure was used to extract nearly all predicted structures using in-house scripts (available upon request). During extraction, a new z-score was calculated for each extracted structure sequence. These structures are referred to as cancer driver -1 (CD-1) structures.

### Determination of MANE transcripts and mapping of UTRs

One transcript per gene was selected (except CDKN2A, where a transcript each for p16^INK4A^ and p14^ARF^ were used) based on the common form of the gene or Matched Annotation from NCBI and EMBL_EBI (MANE) transcript as indicated by Ensembl (Refseq match). A list of MANE transcripts was obtained from NCBI (https://www.ncbi.nlm.nih.gov/refseq/MANE/). If no MANE transcript was listed, a predominant protein-coding form was chosen. A list of gene symbols and Ensembl transcript identifiers (ENSTs) are provided (S1 Table). UTR and coding determinations were made by comparing transcript structure coordinates to a table of UTR coordinates using exon data from Ensembl Biomart (S1 Table).

### Genomic and variant mapping

CD-1 structures were mapped to the human genome (GRCh38) using Bowtie2 (v2.4.2; [19]) to obtain genomic coordinates. These coordinates were used to identify clinical variant mutations (ClinVar; [20]) and COSMIC non-coding mutations (v93; [21]) within the sequences of CD-1 predicted structures (S3 and S4 Tables, respectively). Post-variant mapping, results were corrected by ensuring structures were mapped to the correct chromosome. The matched-sequence mapping was not perfect and it is possible that not all of the predicted structured sequences are included here.

### Covariation model building (CMbuilder) analysis

All CD-1 structures (35,426) were analyzed for covariation using the cm-builder perl script [15, 22]. This script builds off the RNAFramework toolkit [11, 12] and utilizes Infernal (here using release 1.1.2; [22, 23]) to build and search for covariation models from each predicted ScanFold secondary structure. To build a database for Infernal, BLAST from the NCBI Refseq database was performed for each of the 801 MANE reference sequences used in ScanFold with the following parameters:

*$ blastn -db refseq_rna -query "sequence_file*.*txt" -task blastn -out "name_b*.*txt" -gapopen 5 -gapextend 2 -reward 1 -penalty "-1" -outfmt "6 sallgi sallseqid sallacc" -max_target_seqs 2500*
*$ blastdbcmd -db refseq_rna -entry_batch "name_b*.*txt" -out "name_DB*.*txt" -outfmt "%f"*

Perl scripts were then used both to convert the resulting fasta files into single line format and to remove any duplicate sequences. A Python script further narrowed the resulting database down to headers that included the exact gene name of interest and eliminated any pseudogenes. For successful covariation models, the resulting structural alignment files (in Stockholm format) were tested for covarying base pairs and also analyzed with the CaCoFold algorithm using R-scape (version 1.5.16); statistical significance was evaluated by the APC corrected G-test [13, 24] using the default E value of 0.05. All Stockholm alignments and R-scape/CaCoFold results can be found at doi: 10.5281/zenodo.5747774. Expected versus observed covarying base pairs from power files were used to generate a Z-score of CMbuilder (Zcm) for the covariation modeling; Zcm is calculated by taking the difference between the number of observed versus expected covarying pairs divided by the standard deviation of the number of expected pairs (Zcm = (observed—expected) / SD).

### Reporter assays and translational efficiency

Cloning into a modified pmirGLO dual luciferase plasmid (Promega) was done after restriction enzyme digestion with XhoI using the HiFi DNA Assembly kit (NEB) with either gBlocks or Ultramer oligonucleotides (IDT). The pmirGLO modification consisted of introns introduced into each of the firefly and renilla luciferase genes. Sequences were verified using Sanger sequencing (Iowa State University DNA Facility). Empty pmirGLO was the control. HeLa cell (HeLa) transfections were carried out using Lipofectamine 3000 (Invitrogen) into 96-well dishes (5 ng pmirGLO construct, 95 ng pUC19; at least 5 wells each) for reporter analysis and into 24-well dishes (25 ng pmirGLO construct, 475 ng pUC19; 3 wells each) for isolation of RNA. HeLa cells were cultured in DMEM (Gibco) supplemented with 10% FBS (Atlanta Biologicals), penicillin/streptomycin (Gibco), and L-glutamine (Gibco) at 37°C in 5% CO_2_. The overall procedure was as follows: day 0–90–100% confluent cells passaged 1 to 2; day 1 –cells plated (96-well: 20,000 c/well in 100 ul; 24-well: 120,000 c/well in 500 ul); day 2 –transfections; day 3 –cells fed fresh medium (100 ul or 500 ul, respectively); day 4—Dual-Luciferase Reporter Assay System (Promega) carried out (96-well samples) using the GloMax instrument (Promega), and RNA isolated. Relative Response Ratio (RRR) was calculated by dividing the light units from firefly by those of renilla on a per-well basis. This was then normalized to the average of the control and averaged ± standard deviation.

RNA isolation was done using TriZol (Invitrogen) and 1-bromo-3-chloropropane (Sigma-Aldrich) with QuantBio Heavy PLG tubes. The aqueous phase had an equal volume of 100% ethanol added before loading it onto a column from the Direct-Zol RNA Miniprep Kit (Zymo). The RNA prep followed the manufacturer’s instructions with the exception that the on-column DNAse was carried out for 40 minutes. RNA was stored at -80°C. RNA was quantified and analyzed using a NanoDrop One (Thermo-Fisher). First-strand cDNA synthesis was carried out using 1 ug of RNA with Superscript III (Invitrogen) and random hexamers (IDT) on a SimpliAmp (Thermo-Fisher) instrument. Quantitative PCR was performed with 1 ul of 10X-diluted cDNA, cPrimeTime® primer/probes (IDT) designed to overlap the introduced intron for each of the firefly and renilla luciferase genes (firefly: forward 5′′ –ACAAAACCATCGCCCTGATC– 3′, reverse 5′ –ATCTGGTTGCCGAAGATGG– 3′, probe 5′6-FAM/ACCGCTTGT/ZEN/GTCCGATTCAGTCAT/3′IABkFQ; renilla: forward 5′ –CCTACGAGCACCAAGACAAG– 3′, reverse 5′ –ACCATTTTCTCGCCCTCTTC– 3′, probe 5′SUN/CACGTCCAC/ZEN/GACACTCTCAGCAT/3′IABkFQ), and PrimeTime® Gene Expression Master Mix (10 ul total) on a QuantStudio3 (Thermo-Fisher). Ct values were calculated using the automatic settings of the QuantStudio Design & Analysis desktop software (v1.5.1). The ddCt method was employed with renilla and empty pmirGLO as references to get the average fold expression (2^-ddCt^) and standard deviation. Translational efficiencies were calculated by dividing the normalized RRR by the mRNA expression and propagating the error. T-tests were carried out using values of the per well, normalized RRR values divided by the average mRNA expression value with α at 0.05. Raw data can be found in S1 File.

## Results

**Fig 2:**
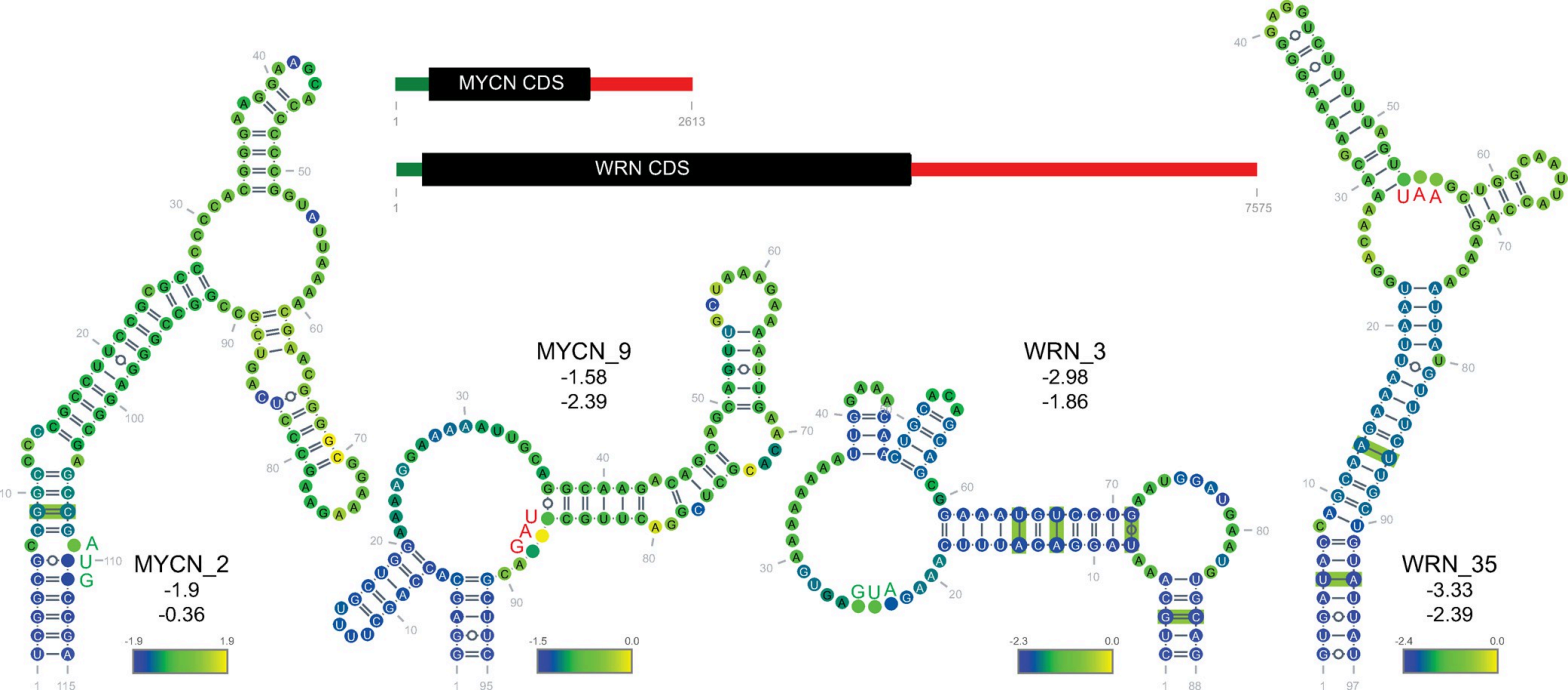
Examples of ScanFold identified structures that overlap the start and stop codons. The neuronal myelocytomatosis (*MYCN*) and Werner’s helicase (*WRN*) transcript diagrams are shown with secondary structures diagramed below. The AUG start codons are shown in green while the stop codons are shown in red. The multi-coloration of the bases represents the per nucleotide z-score mean as generated from ScanFold scanned windows; the scale is indicated. Base pairs boxed in green were found to be covarying after CMbuilder analysis. Values of the structure z-score (not the average of per nucleotide windows) and the Zcm are provided under the structure names, respectively.

To scan for potential functional RNA structural motifs in cancer driver genes, we analyzed data from two different sources. Dietlein et al. (2020) identified 460 potential cancer driver genes through MutPanning. Martinez-Jiménez et al. (2020) identified 568 in the Compendium. Combined, 232 were unique to MutPanning, 340 were unique to the Compendium, and 228 were common to both. We scanned all Ensembl-identified transcripts (10390) for each of these 800 cancer driver genes using ScanFold (Fig 1), identifying 179,190 structures constrained by an original ScanFold z-score of -1 or less (S5 Table). Reducing these structures to one transcript per gene (excepting *CDKN2A*; see Methods) resulted in a total of 35,426 predicted structures with at least one nucleotide exhibiting an average z-score of < -1 after the initial scan (S6 Table). Of these cancer driver -1 (CD-1) structures, about 4% were found in 5′UTR sequences, 52% in coding sequences, and 44% in 3′′UTR sequences. Furthermore, 415 transcripts had structures overlapping either the start codon (233 transcripts) or the stop codon (267 transcripts), with 85 transcripts having structures overlapping both ends of the coding sequence (Fig 2; S7 Table).

**Fig 3:**
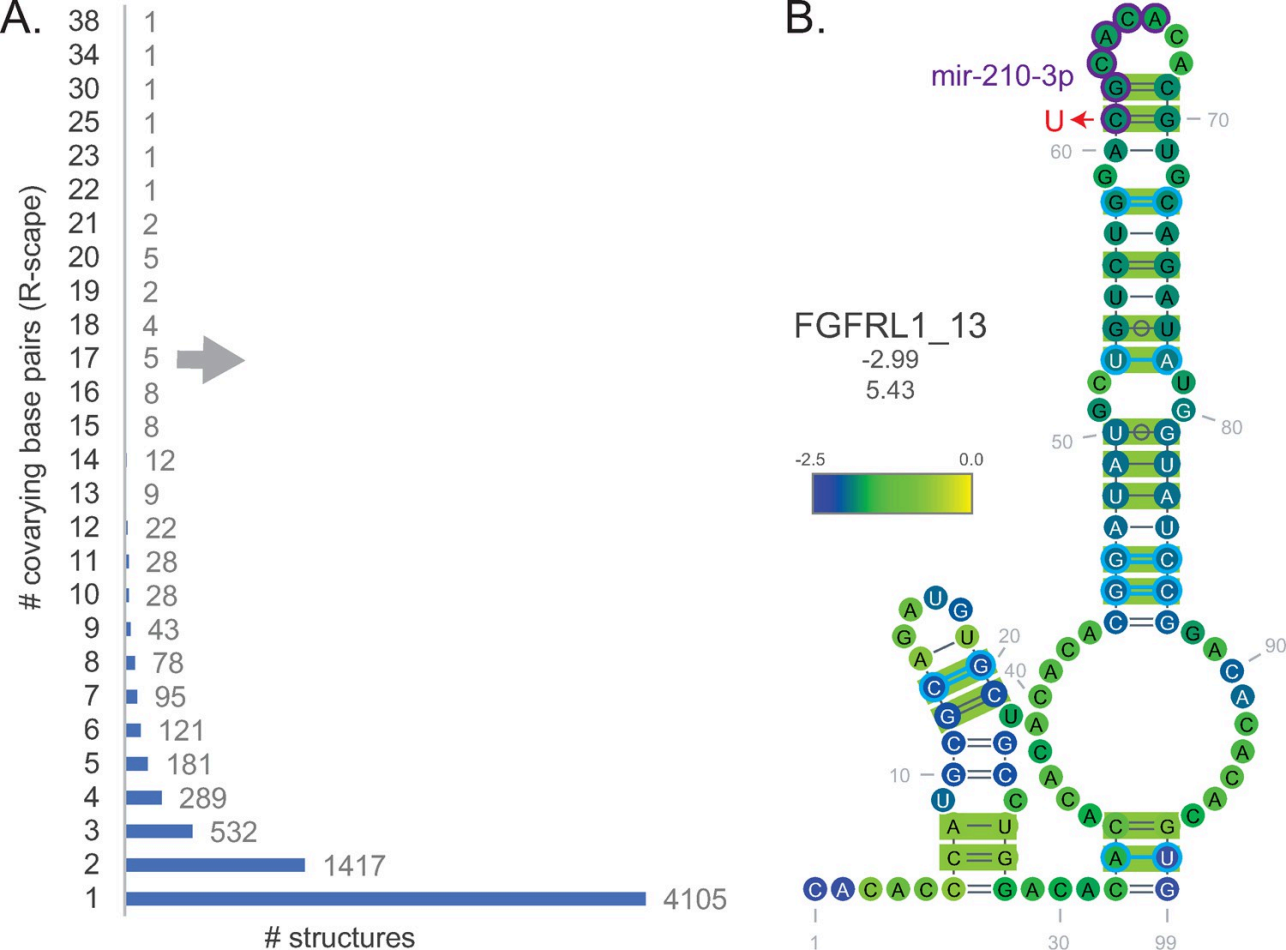
Covariation analysis and example. **(A)** Graph showing the number of structures per the number of covarying base pairs determined by R-scape covariation analysis of all structures. **(B)** Predicted secondary structure of Fibroblast growth factor receptor like 1 structure 13 (*FGFRL1*_13) shown as a middle of the road example with both R-scape (green boxes) and CaCoFold (cyan lines) covariation results. The TargetScan predicted miR-210-3p binding site and an identified COSMIC mutation are marked in purple and red, respectively. Per nucleotide z-score mean is shown as indicated. Values of the structure z-score (not the average of per nucleotide windows) and the Zcm are provided under the structure name, respectively.

Covarying base pairs were observed in three of the start or stop codons that overlap structures shown in Fig 2 (*MYCN*_2, *WRN*_3, *WRN*_35). All CD-1 structures were analyzed for covariation using CMbuilder [12, 15, 22]. By building stringent Infernal alignment databases for each gene–devoid of pseudogenes and matching the gene-name in the header–a high-confidence set of structures that contain covarying base pairs was identified (S8 Table). S2 File provides seven examples of the phylogenetic depth (gene symbol and structure number are indicated at the tops of the trees) based on the nucleotide accession numbers from Stockholm alignment files. R-scape (ScanFold-Fold model structure informed) detected covarying base pairs in 7,001 predicted structures. The majority, 4,105, had only a single covarying pair; however, multiple covarying pairs were detected in other structures (up to 38; Fig 3A). To aid prioritization based on statistics generated by Rscape, a z-score, Zcm, was developed that indicates the number of standard deviations that separate the number of observed versus expected covarying base pairs. Table 1 lists 11 structures with the highest Zcm and at least 5 covarying base pairs. Fig 3A compares the number of structures to the number of covarying base pairs. To highlight an example of a structure with about half the maximum number of identified covarying base pairs: Fibroblast Growth Factor Receptor Like 1 structure 13 (*FGFRL1*_13), located in the 3′UTR, was found to have 17 covarying base pairs (Fig 3B). To further test these results, the program CaCoFold, which uses an orthogonal approach to predict structure based on potential covariation [25] rather than through guidance by ScanFold models, was used. All six of the CaCoFold covarying base pairs (Fig 3B, cyan) were also present in the ScanFold identified structure. Several miR-210-3p binding sites have been identified in the 3′UTR of *FGFRL1*, which is known to reduce FGFRL1 expression [26]. A COSMIC mutation (COSV53257308) in a miR-210-3p seed binding region of *FGFRL1*_13 increased the predicted ED by a factor of two when analyzed by RNA2Dmut [27], indicating a strong mutation-induced structural shift in this 3′UTR region. Notably, *FGFRL1*_13 had over five standard deviations more covarying base pairs than expected (Zcm = 5.43).

**Table 1:** Top 11 structures by Zcm with at least 5 covarying base pairs.

symbol_structure	location	MFE	z-score mean	ED	# covarying	Zcm
**EFTUD2_27**	3′UTR	-14.8	-1.18	2.66	5	25.0
**G**C**AG**CCUCCU**UC**CUGCCUGGGUCCCCAGG**GA**AGCCGC**CU**G**C**						
**(**(**((**.(..((**((**(. . .((((. . . .)))))**))**))..).**))**)**)**						
**USP8_180**	3′UTR	-5.4	-1.38	1.41	8	19.5
**GGC**ACA**UCUAU**AGAAAUAUAUAAA**GUAGA**G**GCC**						
**(((**.(.**(((((**. . . .. . . .. . . ..**)))))**)**)))**						
**AR_8**	5′UTR	-1.7	-0.16	3.93	5	16.3
**GUCUU**CUUCUGCAC**GAGAC**						
**(((((** . . . .. . . .. **)))))**						
**INMT_4**	3′UTR	-24.7	-2.21	0.26	6	14.8
G**C**CUGG**GCCC**UGAGCCAGGA**GGGC**CAGCC**A**GAGGUC**U**GGUCAG**G**C						
(**(**((((**((((**(. . . .. . . .)**))))**).(((**(**(. . . .)**)**))))))**)**)						
**JAK3_29**	3′UTR	-26.4	-5.02	2.00	6	14.5
**GC**C**CU**GGCCCCCUG**AG**UUUCCUUUUCUGUCUCUCUCUUUUUAUUUUUUUUAUUUUUAUUUUUAUUUUUGAGACAGAGCCUCG**CU**CUGUUACCC**AG**G**GU**						
**((**(**((**((. . .. . .(**((**(. . .. . .(((((((((. . . .. . . .. . . .. . . .. . . .. . . .. . . .. . . .. . . .))))))))). . . .)**))**). . .. . .))**))**)**))**						
**SIX1_31**	3′UTR	-16.8	-3.03	6.17	6	14.5
UCUCUG**G**AAAAUA**G**G**A**A**GGG**CCAAUUACUUUAAUUUCUUACAUGCCGU**CUC**U**U**C**C**UACU**C**CGGUGA						
((.(((**(**(. . .((**(**(**(**(**(((**.(. . . .. . . .. . . .. . . .. . . .. . . .).**)))**)**)**)**)**)).)**)**))).))						
**EVI5L_28**	3′UTR	-33.4	-4.44	0.17	7	13.6
C**CC**UCCG**GGCCC**UCUGGCGUUCCAGG**GG**U**GCC**UGGA**GG**G						
(**((**((((**(((((**(((((. . . .)))))**))**.**)))**))))**))**)						
**ZNF233_1**	5′UTR	-33.3	-0.47	25.14	7	13.6
UC**UG**GGAGGUGAG**U**CAGCGCG**G**AACCU**C**UGCAUCUACG**GC**GAGCUUUCCUG**GC**CUGGGCGUUGGACUCGC**A**GUUC**U**GCCUUCC**CA**GG						
.(**((**(((((.(((**(**(((((((**(**(. . .)**)**))).((((.(**((**.((. . . ..)).**))**))))).))).)))))((**(**. . . .**)**)))))))**))**).						
**GLI1_6**	5′UTR	-70.1	-4.01	5.69	18	12.4
**G**G**GCU**G**GG**GGCCAGG**U**U**G**GG**G**G**G**GUG**G**G**GGU**GGCAUCGAG**GC**U**GC**GCUGCC**GU**G**G**C**C**CUCUCC**GCC**C**C**CC**C**U**C**CC**CA**CCGCACACCC**CC**C**AGC**C**C**						
**(**(**(((**(**((**((. . .((**(**.**(**((**(**(**(**(.(**(**(**(((**((. . . .(((**((**(**((**(. . . .)**))**)**)**.**)**))).))**)))**)**)**))**)**)**)**))**))**)). . .. . .))**))**)**)))**)**)**						
**ACVR1B_23**	3′UTR	-23.4	-4.43	2.26	9	12.1
**GGU**GA**U**G**AGA**C**CU**GGGGUUUAGAACCCC**AG**G**U**GAGAC**CU**C**A**AAUC**ACU**						
**(((**((**(**(**(((**(**((**((((((. . .))))))**))**)**)**. . . ..**))**)**)**..))**)))**						
**ARL16_14**	3′UTR	-17.1	-3.86	1.48	5	12.0
**UA**GCUGGGUG**UGC**UGGUGCAUGCCU**GUA**AUCUCAGC**UA**						
**((**((((((..**(((**.(((. . . .))).**)))**..))))))**))**						

**Fig 4:**
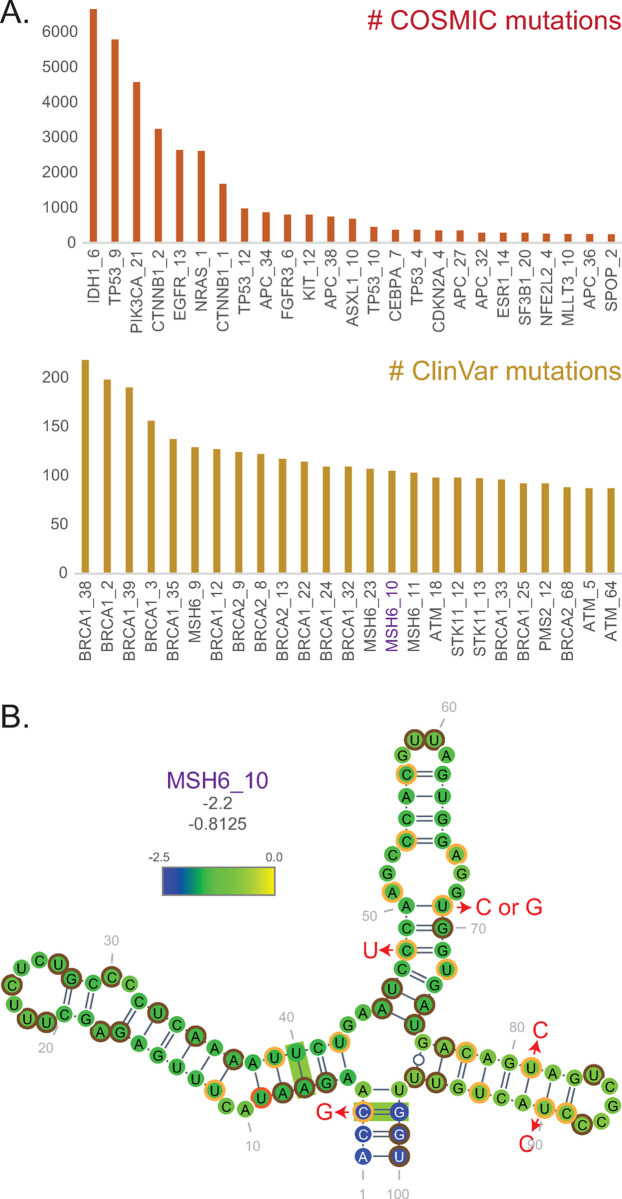
Structure-filtered mutations. Mutations found within ScanFold extracted structures were mapped back to the genome prior to mapping variants from the COSMIC non-coding database [21] and from ClinVar [20]. **(A)** 25 most frequently mutated structures from each of COSMIC and ClinVar are shown. **(B)** *MSH6*_10 (Musashi homolog 6 structure 10) is highlighted because it contains the lowest Zcm of structures from A that have at least one covarying base pair (green boxes; R-scape). The only nucleotides that do not have a reported CinVar mutation are circled in brown, whereas those circled in orange have reported synonymous mutations. Arrows point from nucleotides to reported mutations (red) that resulted in destabilizing changes in ED of four or greater after analysis with RNA2Dmut [27] (S1 Fig). Bases are colored according to the per nucleotide z-score mean as before. Values of the structure z-score (not the average of per nucleotide windows) and the Zcm are provided under the structure names, respectively.

To make comparisons to available genomic variation data, the sequences corresponding to predicted CD-1 structures were mapped to the human genome using Bowtie2 [19]. The genomic coordinates (GRCh38) were used to identify predicted CD-1 structures that contain COSMIC non-coding variants or ClinVars (S3 and S4 Tables). For COSMIC, 141,221 variants were mapped to 11,740 structures in 767 genes, whereas 36,255 ClinVars were mapped to 6,115 structures in 545 genes. Fig 4A shows data for the 25 structures with the most reported variants (not length adjusted). Many of these structures contribute heavily to the number of variant-containing structures per gene (S1A Fig). Musashi homolog 6 structure 10 (*MSH6*_10) was chosen as an example because it has the lowest Zcm of the top 25 structures that had at least one covarying base pair (Fig 4B). Though found in a coding region, it also sustained many synonymous mutations. To query potential effects of these, the sequence was analyzed using RNA2Dmut [27] (S1B Fig). Six of the synonymous mutations increased the predicted ensemble diversity (ED; a measure of different potential conformations–the lower the more likely a single conformation predominates) by at least four (Fig 4B). Two of these, structure positions 69 (T>G) and 81 (T>C) (ClinVars 818324 and 743181, respectively), resulted in a *four-fold* increase in ED, suggesting that these mutations have high potential for disrupting secondary structure.

**Fig 5:**
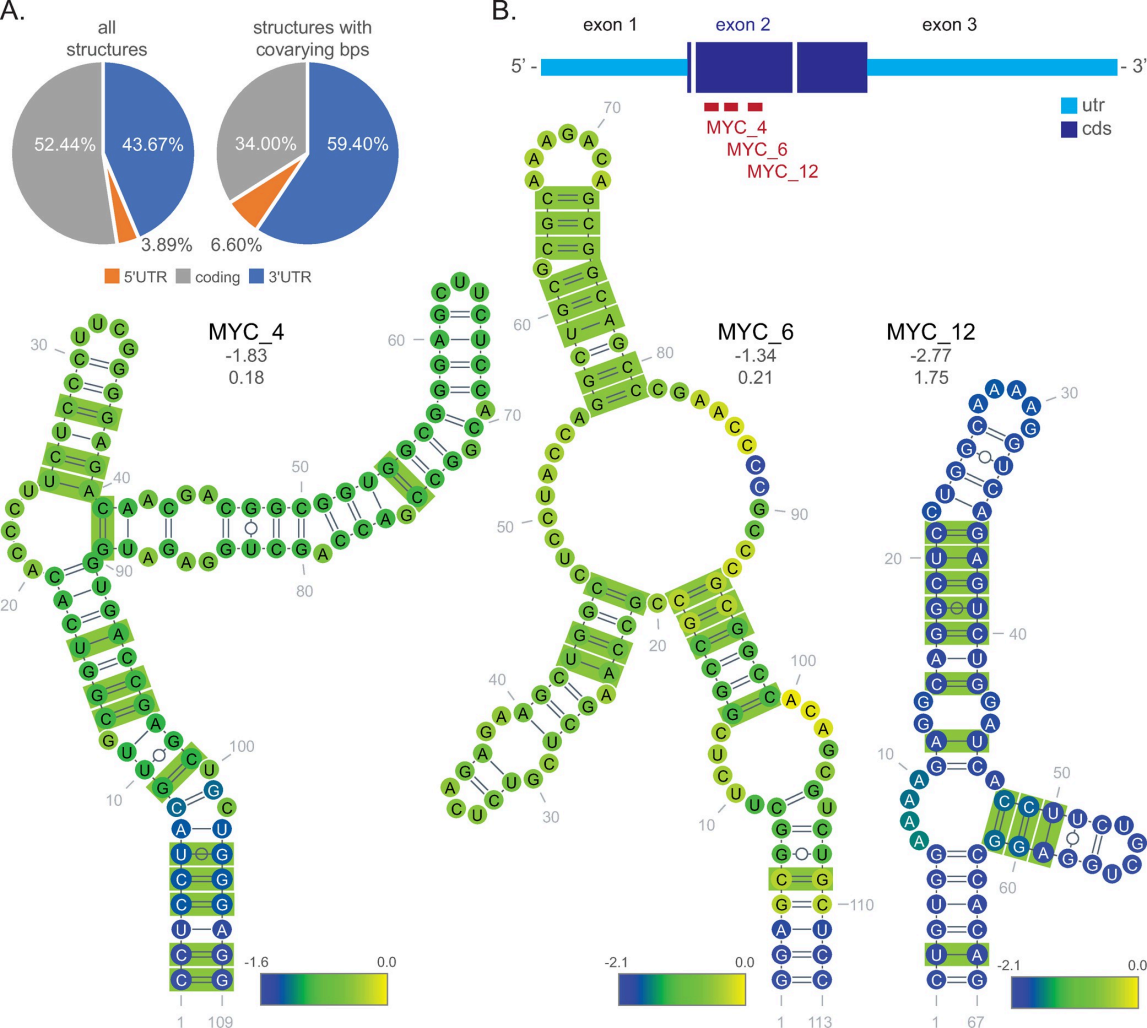
Location of structure analysis and coding structure examples. **(A)** Graph of UTR and coding region percentages across all identified structures (left) and the subset of structures that had at least one covarying base pair (right). **(B)** Schematic of *MYC* transcript showing the location of three conserved structures in exon 2. Secondary structures are shown below with covarying base pairs denoted in green boxes (R-scape). Base coloring indicates per nucleotide z-score mean. Values of the structure z-score (not the average of per nucleotide windows) and the Zcm are provided under the structure names, respectively.

*MSH6*_10 also contained covarying base pairs (green boxes, Fig 4B), indicating that the base pairing has been preserved through mutational events in evolution. Structures with covariation were found in all mRNA regions, but with more in the UTRs, proportional to the number of total identified structures (Fig 5A; mean lengths: 5’UTR = 258, coding = 3,136, 3′UTR = 2,306). Interesting structures in coding regions were, however, still predicted. For example, three structures encoded in exon 2 of the Myelocytomatosis gene (*MYC*) each had 10 covarying base pairs (Fig 5B); a covarying base pair found on each of the predicted stem loops in each of these multi-branch structures lends support to the structure models. Interestingly, a larger region that encompassed structures *MYC*_4 and *MYC*_6 was previously found to confer downregulation of *MYC* mRNA during induced myoblast differentiation [28, 29] Whether these structures in particular play a role in the destabilization of the *MYC* transcript remains to be determined.

**Fig 6:**
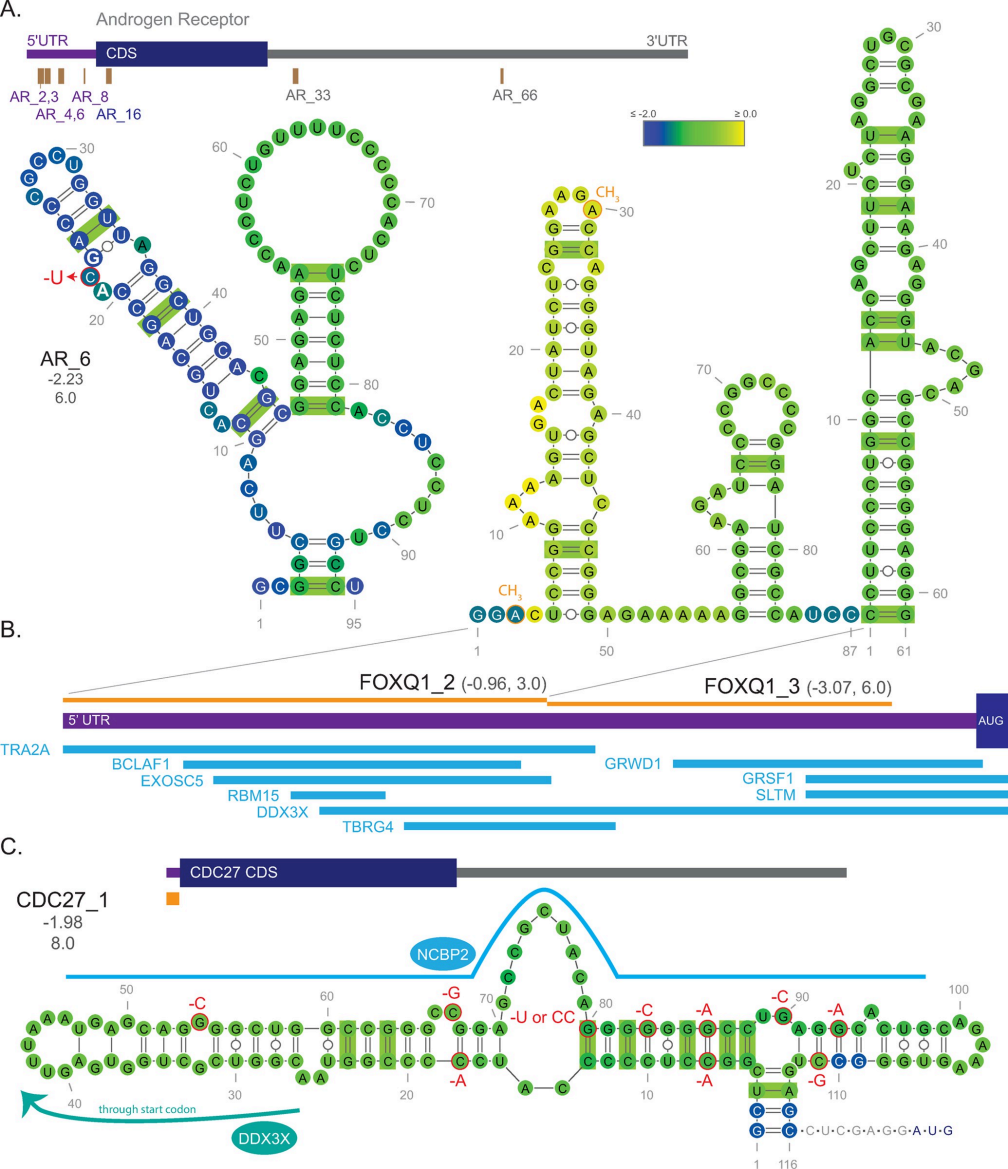
Examples of 5′UTR structures. Covarying base pairs are denoted using green bars (R-scape). **(A)** Schematic showing structured regions of the androgen receptor (AR) that had at least 5 covarying base pairs. Below the transcript schematic is structure 6; the upstream open reading frame (uORF) inducing mutation that can cause Complete Androgen Insensitivity Syndrome is shown in red. **(B)** Forkhead box Q1 (FOXQ1) 5′UTR schematic showing two adjacent structures (2 and 3) and the proteins determined to bind in these structured regions (blue) by eCLIP. C. Secondary structure of Cell division cycle 27 structure 1 extended to show the distance to the start codon. Reported COSMIC mutations are shown in red and the eCLIP binding region of nuclear cap binding protein 2 (NCBP2) is shown in blue. The average z-score of all the shown nucleotides in the structure is shown directly below the name of the structure with the Zcm below that. Base coloring indicates per nucleotide z-score mean as indicated by the scale. Values of the structure z-score (not the average of per nucleotide windows) and the Zcm are provided under the structure names, respectively.

Many interesting 5′UTR discoveries were made (S8 Table). For example, the chromatin remodeling factor Special AT-rich sequence binding protein 1 (*SATB1*) had three structures (_8, _15, _19) in the 5′UTR with over 10 covarying base pairs. Eight Androgen Receptor (AR) structures were found throughout the transcript—each contained five or more covarying base pairs, including structure 6 (AR_6) that overlaps a mutationally-induced upstream open reading frame (uORF) in its 5′UTR (Fig 6A). When this mutation occurs, Complete Androgen Insensitivity Syndrome (CAIS) can result from production of an uORF which ablates AR protein production [30]. Intriguingly, the C>U mutation that generates the uORF is predicted to form a stabilizing UA base pair, further strengthening the predicted conserved hairpin structure in which it falls.

Additional examples of conserved RNA structures in 5′′UTRs can be seen in Forkhead box Q1 structures 2 and 3 (*FOXQ1*_2, _3), which are adjacent to one another and are just 16 nucleotides away from the start codon. Cross-referenced enhanced crosslinking immunoprecipitation (eCLIP) studies found through ENCODE [31] revealed many potential regulatory RNA-binding proteins (Fig 6B) overlapping these predicted structures. Notably, the 5′UTR of *FOXQ1* mRNA is methylated in the first hairpin’s terminal tetraloop adjacent to the binding region of the methyltransferase RBM15, which binds at the base of that stem loop. This region overlaps the binding region of TRA2A, which has been shown to enhance methylation of mRNA in association with methytransferases [32]. Another potential connection involving TRA2A is that both it and FOXQ1 activity have been implicated during the cancer progressing epithelial to mesenchymal transition (EMT) [33–35], as has the DEAD (Asp-Glu-Ala-Asp)-box helicase 3X (DDX3X) protein that was also found to bind in the same region of *FOXQ1* mRNA [36].

A final example of a predicted conserved 5′UTR structure is anaphase promoting complex subunit Cell Division Cycle 27 (*CDC27*_1) that was found just seven nucleotides away from the start codon and contained eCLIP-identified binding sites for nuclear cap binding protein 2 (NCBP2; also CBP20) and DDX3X (Fig 6C). NCBP2 is part of the cap-binding complex responsible for translational control and monitoring mRNA integrity [37] and association with DDX3X on 5′UTRs has been shown to drive uORF translation [38]. A potential uORF of CDC27 that begins in the loop of the long hairpin would result in a premature stop codon just prior to the start codon, likely disrupting CDC27 translation. Notably, this 5′UTR region contains many clinically relevant variants, with 11 different mutations found in cancers. All but four mutations occurred in base paired sites, where they are predicted to disrupt structure (S3 File).

**Fig 7:**
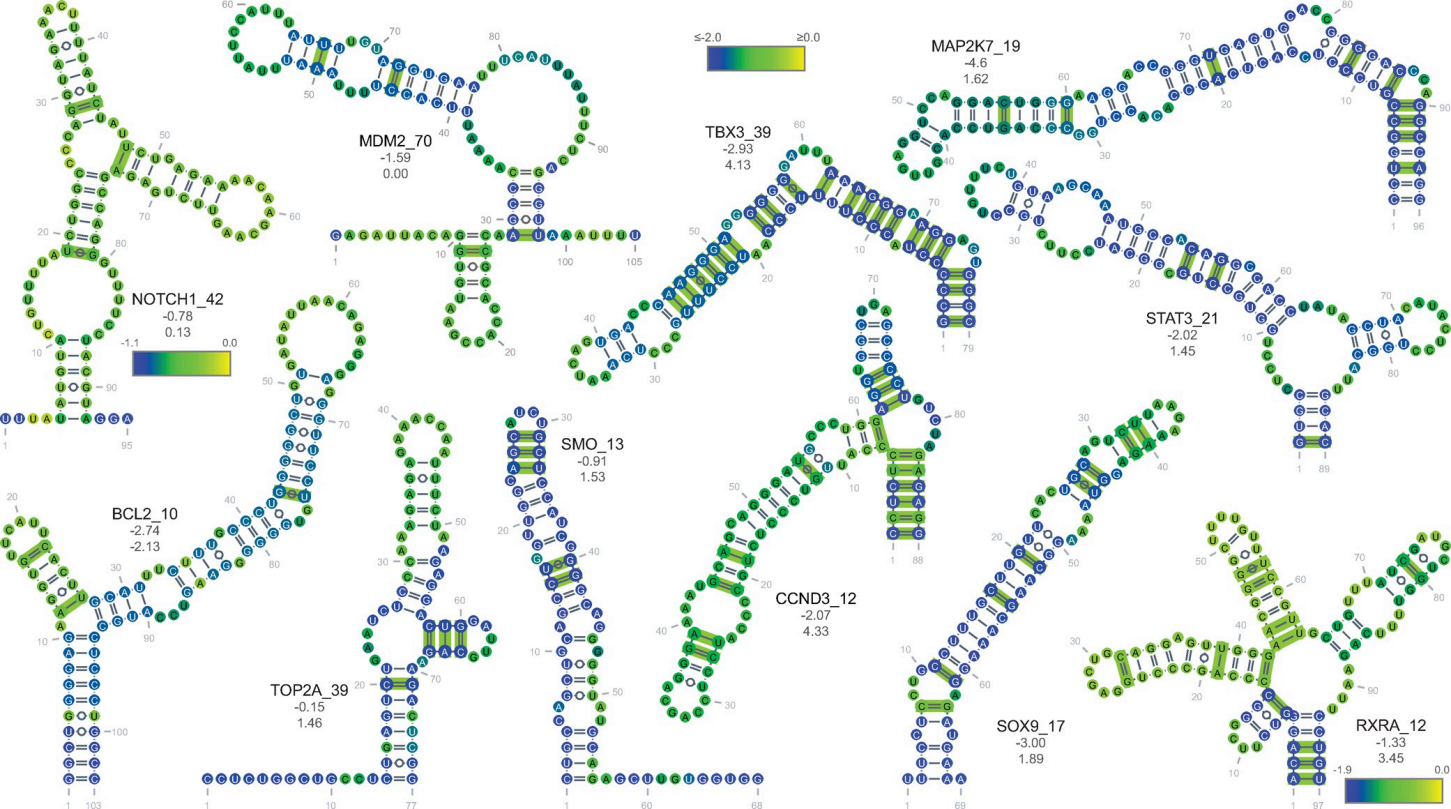
Examples of 3′UTR structures. Covarying base pairs are denoted in green (R-scape). The gene symbol and structure number are shown next to the secondary structure diagram. Zscore average per structure indicated below the name with the Zcm below that. Scale indicates per nucleotide average z-score for all calculated windows. The structure z-score (not the average of per nucleotide windows) and the Zcm are provided under the structure names, respectively.

As mentioned above, the greatest number of structures were predicted in 3′UTRs (Fig 5A; S6 and S7 Tables). To highlight several example classes: genes encoding receptors, such as the Retinoid X Receptor Alpha (*RXRA*), Notch receptor 1 (*NOTCH1*) and Smoothened, frizzled class receptor (SMO), signaling kinases, such as Mitogen-activated protein kinase kinase 7 (*MAP2K7*), E3 ubiquitin ligases, such as Murine Double-Minute 2 (*MDM2*), anti-apoptotics, such as B cell Lymphoma 2 (BCL2), isomerases, such as Topoisomerase 2 alpha (*TOP2A*), cyclins, such as Cyclin D3 (*CCND3*), and transcription factors, such as Signal Transducer and Activator of Transcription 3 (*STAT3*), SRY-box transcription factor 9 (*SOX9*) and T-box transcription factor 3 (*TBX3*), all showed conserved structures (Fig 7). It is noteworthy that these highlighted examples, represent just a fraction of the structures identified. Table 2 lists several features that overlap these structure regions, including eCLIP-identified binding proteins, TargetScan-predicted miRNA binding, the presence of COSMIC or ClinVar mutations and methylation of adenosine.

**Table 2:** Fig 7 structures with overlapping sequence features as indicated.

	eCLIP		miRNA	mutations	# methyl-adenosine
**BCL2_10**				2 COSMIC SNPs	
**CCND3_12**	DHX30	FAM120A	miR_1306-5p		
	HNRNPK	PABPC4			
	PCBP1	SF3B1			
	SUPV3L1	UPF1			
	UTP3	ZC3H11A			
**MAP2K7_19** *	AKAP1	AKAP8L			3
** **	CDC40	CSTF2T			
** **	DDX24	DDX6			
** **	DHX30	DROSHA			
** **	EWSR1	FAM120A			
** **	FUS	GRSF1			
** **	GTF2F1	PABPC4			
** **	PABPN1	PRPF4			
** **	PUM2	RBFOX2			
** **	SDAD	SUB1			
** **	UPF1	XRN2			
** **	ZC3H11A				
**MDM2_70**				1 COSMIC SNP	
**NOTCH1_42**	DDX6	TIA1			2
**RXRA_12**	AKAP1				
**SMO_13**					
**SOX9_17**	DDX6	UPF1		ClinVar indel—Camptomelic dysplasia	
				2 COSMIC SNPs	
**STAT3_21**	AKAP1	FAM120A	miR-17-5p	ClinVar SNP -Hyper-IgE recurrent infection syndrome 1, autosomal dominant	
** **	SUB1	UPF1	miR-93-5p		
** **			miR-106-5p		
** **			miR-519-3p		
** **			miR-130-3p		
** **			miR-301-3p		
** **			miR-454-3p		
** **			miR-655 (x2)		
**TBX3_39**	AKAP1 DDX6	DDX55 UPF1		3 ClinVar SNPs -ulnar mammary syndrome	
**TOP2A_39**	DDX55	DDX6			3
** **	IGF2BP1	IGF2BP2			
	IGF2BP3	SUB1			
	TARDBP				

* all on the 5′ half except for UPF1 and FAM120A that ALSO have binding regions on the 3′ half.

**Fig 8:**
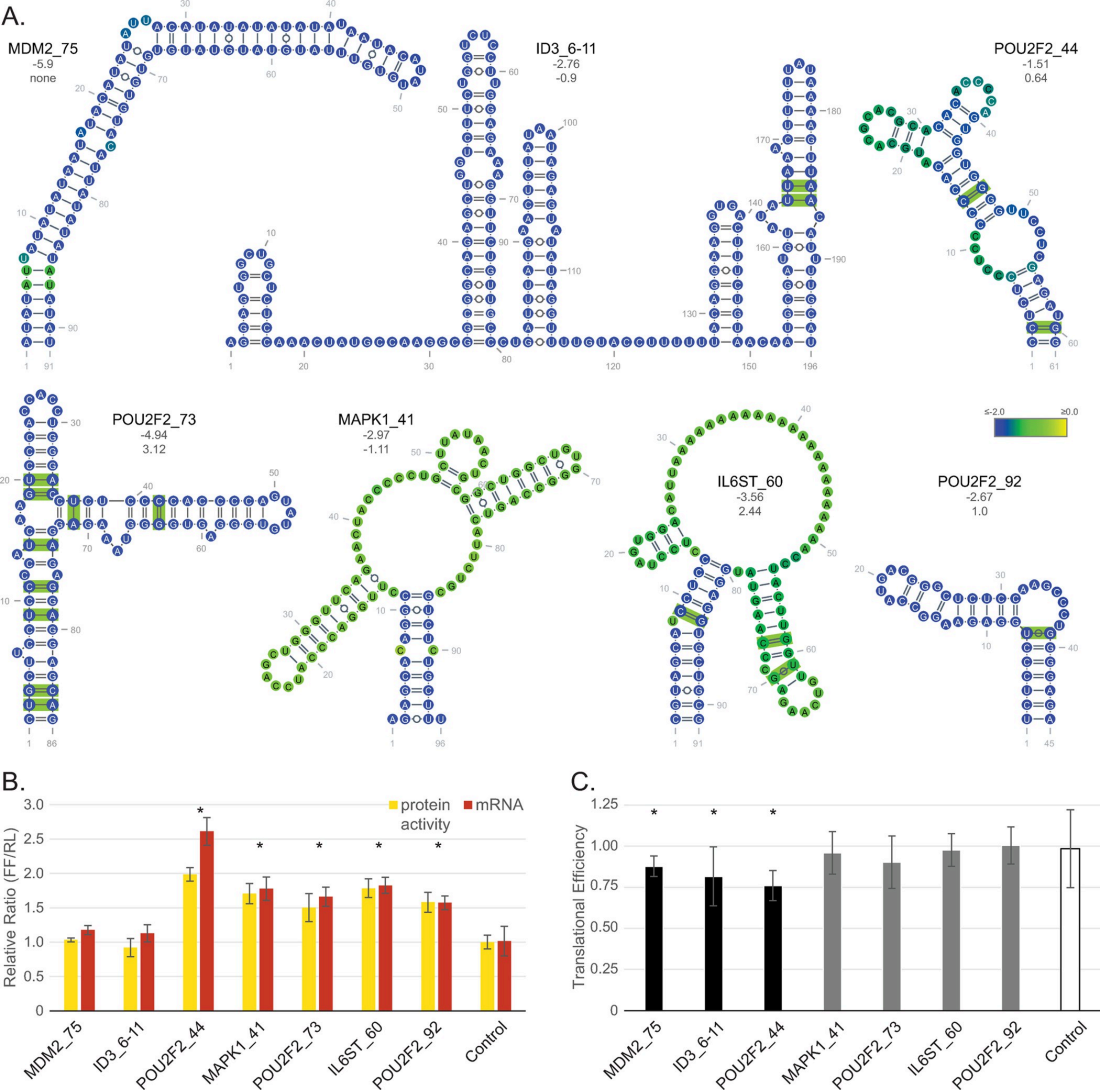
Reporter assay showing effects of 3′UTR structures on translation. **(A)** Secondary structures of the seven sequences cloned and tested in a dual luciferase assay. Covarying base pairs are denoted in green (R-scape). Structure average z-scores and Zcm numerics indicated below the name. Colored bases indicate the z-score average per nucleotide for all calculated windows. **(B)** Graph showing the results of both the dual luciferase assay (yellow; RRR; n = 5 or 6) and corresponding ratio of firefly to renilla mRNA expression (red, n = 3). **(C)** Graph of the translational efficiency (RRR/mRNA) using data from B. Asterisks denote a p < 0.05 by t-test to control. The structure z-score (not the average of per nucleotide windows) and the Zcm are provided under the structure names, respectively.

The potential implications of the structures and their associated features are many. The data generated in this study represents a deep reservoir of information to drive hypotheses generation. To highlight how the data can be used, we chose a variety of predicted 3′UTR structures to test in luciferase reporter assays due to the simplicity of the functional readouts–protein activity and transcript quantitation. We selected targets from a range of different genes where we intentionally picked putative oncogenes with varying levels of covariation support (Fig 8A). *MDM2*_75, *POU2F2*_44 (POU Class 2 Homeobox 2 transcription factor) and *MAPK1*_41 (Mitogen Activated Protein Kinase 1) lacked any evidence of covariation. *MDM2*_75 is found in the longest transcripts that code for the TP53 antagonist within a region just upstream (9 nt) from a putative HNRNPC binding site and is modeled to form a long tetraloop hairpin structure. *MAPK1* encodes the serine/threonine kinase ERK2, a major component of MAP kinase signaling downstream of RAS [39]. *MAPK1*_41 is located toward the 3′-most end of the 3′UTR, is a potential binding site for FAM120A, and has a multibranch structure. *POU2F2*_44 represented another multibranched model structure. Originally identified as a B-cell specific transcription factor, POU2F2 has also been implicated in several other cancer cell lineages as well [40–42]. *POU2F2[29]*_44 was the middle sized of the chosen *POU2F2* structural motifs and closest to the coding region (1461 nt away). None of these have known RNA binding proteins. The shortest sequence tested (45 nt) was the pentaloop hairpin structure *POU2F2*_92, which contained a single covarying base pair and was the only structure represented where each nucleotide in it had a z-score average of > -2. The largest sequence region tested encompassed predicted motifs 6 to 11 from the Inhibitor of DNA binding/differentiation 3 (*ID3*_6–11; 196 nt). Like POU2F2, the transcription factor ID3 is involved in both cell proliferation and differentiation [43]. *ID3*_6–11 contained two covarying base pairs in two of the five predicted hairpins that all had either terminal tri- or tetra loops. Toward the end of a short (515 nt) 3′UTR, this structure encompasses a region with many eCLIP discovered binding proteins (DDX6, DDX55, LARP4, PABPC4, PUM2, TIA1, UPF1) and three predicted miRNA binding sites. With three covarying base pairs, two of the three modeled Interleukin 6 Cytokine Family Signal Transducer structure 60 (*IL6ST*_60) helices are strongly supported. *IL6ST* encodes GP130 which binds the IL6 and IL6 receptor complex, among other cytokine and receptor combinations, to facilitate downstream intracellular signaling for protective immunity and development [44]. Overactive IL6 is a characteristic of B cell neoplasms and conditions such as Multicentric Castleman’s disease [45]. Finally, *POU2F2*_73 contained three times the number of covarying bases as *IL6ST*_60 (nine). This branched structure modeled with two terminal pentaloops and is found devoid of known miRNA or RNA binding protein interactions.

Irrespective of the level of covariation support, each ScanFold predicted region showed activity when inserted downstream of luciferase. Under our conditions, sequences from *POU2F2* (all), *MAPK1* and *IL6ST* stabilized the firefly luciferase mRNA leading to increases in luciferase activity (Fig 8B) compared to the control. The level of enzyme activity in the presence of the *POU2F2*_44 3′′UTR sequence did not, however, match the increase in mRNA, resulting in a decreased translational efficiency (Fig 8C). Modest but statistically significant reductions in translational efficiency were also observed for sequences from *MDM2* and *ID3* despite the lack of difference at the mRNA or reporter activity levels. These results demonstrate how using ScanFold can help identify sequences that have functional outcomes in a reporter assay.

## Discussion

We have predicted structured regions in cancer-related mRNAs and have determined whether these regions are evolutionarily significant through covariation. The strength of our predicted data is supported by the small, diverse (e.g., gene function, sequence, and modeled structure) panel of 3′UTR structures tested in reporter assays. To date, these are the only predicted structures we have tested from this study. We demonstrate that all of these various sequences with predicted structures have a functional impact on the stability of mRNA or on translational efficiency. Importantly, these data support the ability of ScanFold to predict functional sequence regions even in the absence of covarying base pairs.

The strength of our ScanFold-based approach is to identify regions that are most likely to have functional structures. The example modeled structures highlighted here represent just a fraction of the total number we identified– 7000 with covariation support alone. Determining the exact structure and function will require other methods, such as structure probing (to help place them in wider structural context of their respective transcripts), high resolution methods (to deduce their tertiary structures) and CRISPR-based approaches to understand their functional impacts under different cellular environments/contexts/conditions. Nevertheless, these data are an important resource and springboard for hypotheses generation and testing, providing a starting point toward understanding whether targeting conserved functional structures of RNA may yet prove to be an Achilles’ heel to cancer.

Though long considered an untenable option, recent efforts have seen the development of small molecule drugs that target and inhibit RNA structured regions [17, 46]. Though many effective cancer therapeutics target cancer-related proteins, unfortunately such a targeting strategy is plagued by the outgrowth of cells that have undergone mutational selection to render a drug-resistant, relapsed cancer. As the understanding of RNA structure and function increases, the ability to tailor treatments toward mRNA will increase, including targeting of cancer-associated RNA [47]. Targeting RNA has potential to provide a path to mitigate drug resistance, perhaps through simultaneous treatment that targets both the protein and its mRNA–protein-targeting may compensate for mRNA-targeting that is less than 100% effective, and vice versa.

In summary, this study is the first to provide modeled RNA structure covariation data on 800 genes of interest to cancer biology. The data herein are all publicly available and should serve as a valuable resource for the community.

## Supporting information

S1 Table Initial cancer driver gene list, study in which it was identified, matched annotation between NCBI and Ensembl (MANE) identifiers (Ensembl transcript ID), UTR coordinates, and other information. (XLSX)

S2 Table List of transcripts scanned using ScanFold. (XLSX)

S3 Table ClinVars mapped to z-score ≤ -1 cancer driver sequences. (TXT)

S4 Table COSMIC non-coding variants mapped to z-score ≤ -1 cancer driver sequences. (TXT)

S5 Table Z-score ≤ -1 predicted structures identified for all Ensembl cancer driver transcripts. (TXT)

S6 Table Z-score ≤ -1 predicted structures for all MANE cancer driver transcripts. (TXT)

S7 Table Z-score ≤ -1 predicted structures that overlap either start or stop codons for the indicated transcript. (TXT)

S8 Table Z-score ≤ -1 predicted structures after CMbuilder analysis with numbers of expected (± standard deviation) and observed covarying base pairs and associated Zcm score. (TXT)

S1 File Reporter assay raw data. (XLSX)

S2 File Phylogenetic tree-depth of selected predicted structure sequences. (TXT)

S3 File CDC27 structure 1 RNA2Dmut mutational analysis. (XLSX)

S1 Fig ScanFold extracted structures mapped back to the genome prior to mapping variants from the COSMIC non-coding database and ClinVar. **(A)** 25 most frequently represented mutated genes (by number of structures per gene) from each of COSMIC and ClinVar are shown. **(B)** RNA2Dmut outputs for MSH6_10 (Musashi homolog 6 structure 10), represented because it contains the lowest Zcm of the most represented structures that have at least one covarying base pair (green boxes; Rscape). Nucleotides circled in orange have reported synonymous mutations. Bases are colored according to the change in ensemble diversity (ED). The Min (blue scale) represents a reduction in ED or potentially stabilizing effect on the structure. The Max (red scale represents an increase in ED or potentially destabilizing effect on the structure. Values of the structure z-score and the Zcm are given under the structure name, respectively.
(TIF)
