# Supplementary figures and images for: Analyses of human cancer driver genes uncovers evolutionarily conserved RNA structural elements involved in posttranscriptional control
Source: PLoS One. 2022 Feb 25;17(2):e0264025. doi: 10.1371/journal.pone.0264025 (PMC8880891; doi:10.1371/journal.pone.0264025)

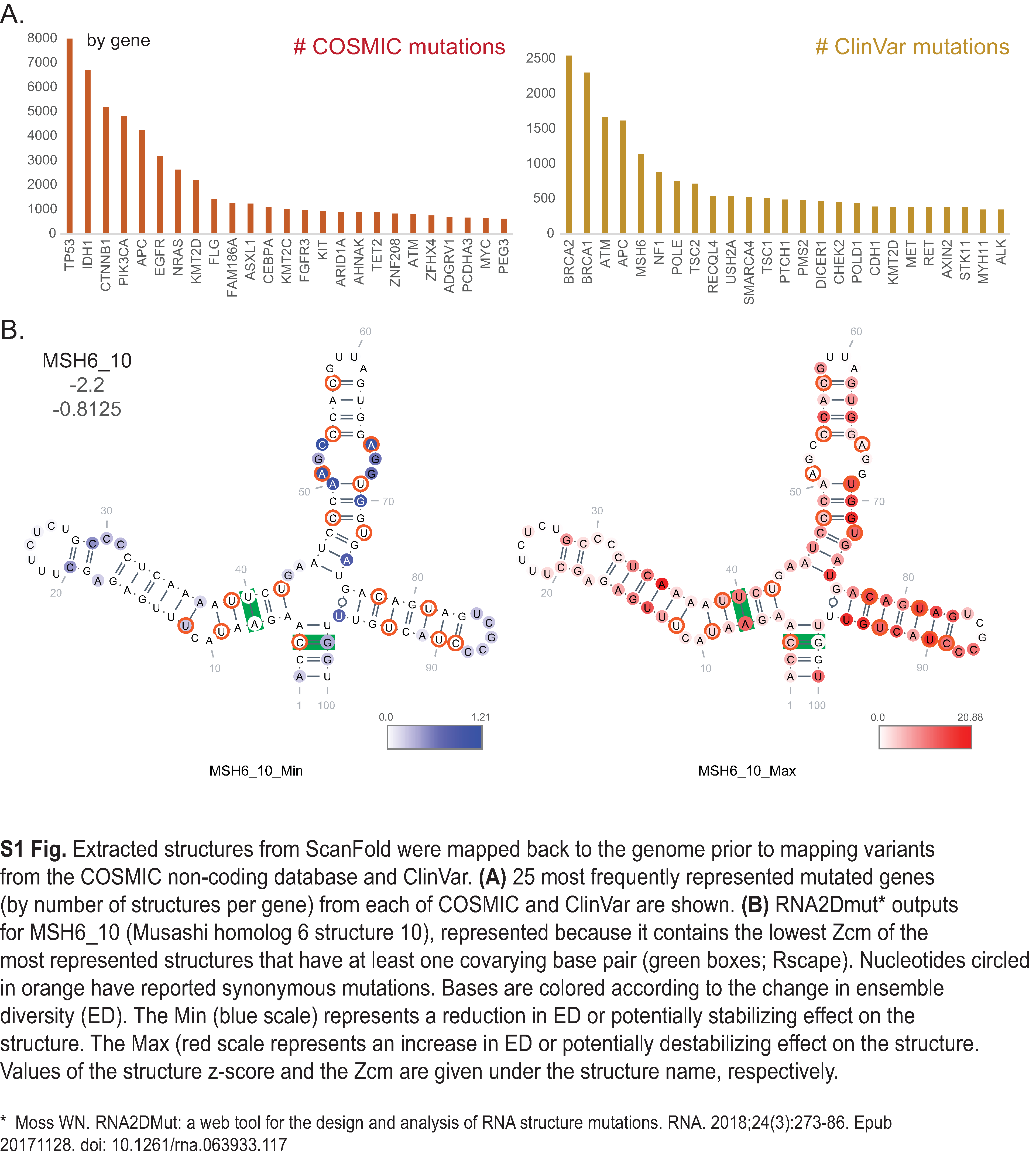

Supplement: S1 Fig — (A) 25 most frequently represented mutated genes (by number of structures per gene) from each of COSMIC and ClinVar are shown. (B) RNA2Dmut outputs for MSH6_10 (Musashi homolog 6 structure 10), represented because it contains the lowest Zcm of the most represented structures that have at least one covarying base pair (green boxes; Rscape). Nucleotides circled in orange have reported synonymous mutations. Bases are colored according to the change in ensemble diversity (ED). The Min (blue scale) represents a reduction in ED or potentially stabilizing effect on the structure. The Max (red scale represents an increase in ED or potentially destabilizing effect on the structure. Values of the structure z-score and the Zcm are given under the structure name, respectively. (TIF) [file pone.0264025.s012.tif]
